# Supplementary figures and images for: Effect of environmental variables on the abundance of Amblyomma ticks, potential vectors of Rickettsia parkeri in central Brazil
Source: PLoS One. 2024 May 15;19(5):e0301685. doi: 10.1371/journal.pone.0301685 (PMC11095677; doi:10.1371/journal.pone.0301685)

## Slide 1
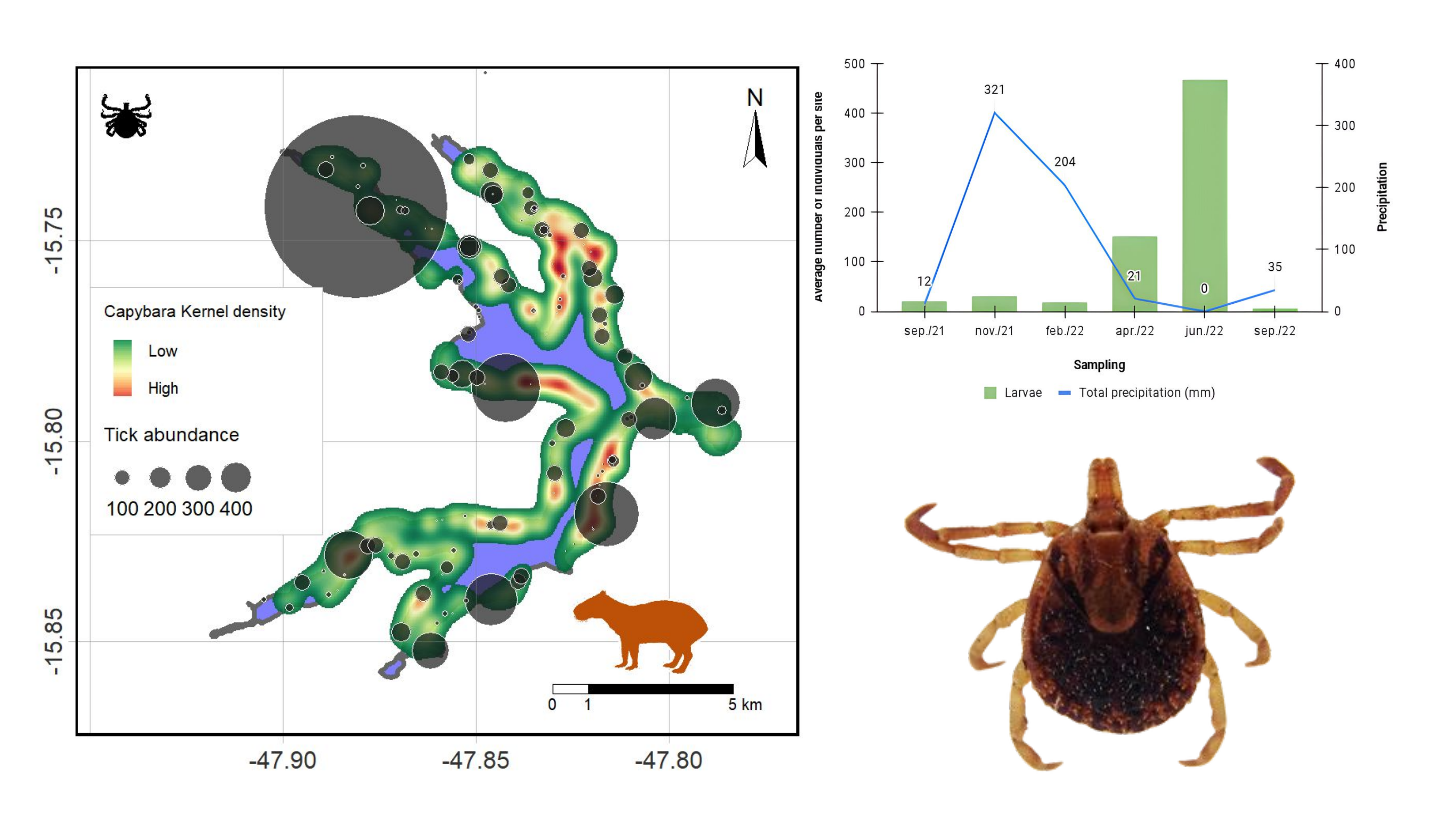

Supplement: S1 Graphical abstract — (PPTX) [file pone.0301685.s004.pptx]
