# Supplementary material for: Effect of environmental variables on the abundance of Amblyomma ticks, potential vectors of Rickettsia parkeri in central Brazil
Source: PLoS One. 2024 May 15;19(5):e0301685. doi: 10.1371/journal.pone.0301685 (PMC11095677; doi:10.1371/journal.pone.0301685)
Supplement: Attachment — Submitted filename: Rebuttal letter_Ticks final 3.docx [file pone.0301685.s005.docx]

PLOS ONE

We've checked your submission and before we can proceed, we need you to address the following issues:

1. Thank you for updating the Figure captions. Before we can proceed, please respond to the following final prompts:

a) Please confirm whether the material from MapBiomas Brasil has been removed from the manuscript and all associated figures.

b) The URL provided for the IPEDF material, <https://na01.safelinks.protection.outlook.com/?url=https%3A%2F%2Fcatalogo.ipe.df.gov.br%2Flayers%2Fgeonode_data&data=05%7C02%7C%7Cfe667946fd3d430e858808dc42aef612%7C84df9e7fe9f640afb435aaaaaaaaaaaa%7C1%7C0%7C638458566428349753%7CUnknown%7CTWFpbGZsb3d8eyJWIjoiMC4wLjAwMDAiLCJQIjoiV2luMzIiLCJBTiI6Ik1haWwiLCJXVCI6Mn0%3D%7C0%7C%7C%7C&sdata=NrFIFcJjY4lQReoRv4ghf1lGXApmMkG8cbru7fCzmDY%3D&reserved=0:> geonode :Lagos_e_reservatorios, is not functional for the journal office. However, we were able to locate a similar URL which may be the same map: <https://na01.safelinks.protection.outlook.com/?url=https%3A%2F%2Fcatalogo.ipe.df.gov.br%2Flayers%2Fgeonode_data%3Ageonode%3ALagos_e_reservatorios&data=05%7C02%7C%7Cfe667946fd3d430e858808dc42aef612%7C84df9e7fe9f640afb435aaaaaaaaaaaa%7C1%7C0%7C638458566428363412%7CUnknown%7CTWFpbGZsb3d8eyJWIjoiMC4wLjAwMDAiLCJQIjoiV2luMzIiLCJBTiI6Ik1haWwiLCJXVCI6Mn0%3D%7C0%7C%7C%7C&sdata=JAzVi9qniTlW0q5FiwrXfdDtAm5Ja3Jt7gS11lBupOw%3D&reserved=0>. If this second URL does indeed link to the correct map, please update the captions to use this URL instead, or else another functional URL.

**Authors:**

**a) yes, we have removed all the material from MapBiomas Brasil.**

**b) we will use the suggested URL which has been included in the caption of the figure in the new version.**

**Reviewer #1:** The paper is well written. The methods were sufficient to answer the questions and the discussion is in accordance with the results obtained.
I have just one comment:

Lines. 283-284. “...Surprisingly, the presence or vestige of capybaras showed no effect on the number of ticks of all life stages...”

The authors completely ignored the fact that Lake Paranoá (in Brasília) is surrounded by a highly urbanized matrix. Anyone who reads the manuscript and doesn't know Brasília might even think that the study was carried out on the edge of a peri-urban lake, which is definitely not the case. The lack of relationship between the number of capybaras and the abundance of ticks in the sampled region is most likely related to urbanization. Most likely, the number of ticks may be related more to the number of domestic animals (especially dogs and cats) than specifically to capybaras. It is true that capybaras are the primary hosts of A. dubitatum, however, the same cannot be said for A. sculptum. It has been demonstrated that A. sculptum also parasitizes dogs and cats, including ticks infected with Rickettsia (see: Mendes et al. 2019 and Campos et al. 2020). Lake Paranoá, in addition to being surrounded by luxurious mansions and farms, whose owners raise many dogs and cats, residents from other regions of Brasília take their pets to the place for leisurely walks. The presence of these domestic animals in the research region, in my opinion, should not have been ignored in the experimental design. As it is not possible to return to the data collection phase, I suggest that the authors discuss whether urbanization and the presence of dogs and cats in the sampled area would have any impact on the results achieved.

Authors: We appreciate the reviewer's comment. We have detailed the study area in lines 88-90 by clarifying that Lake Paranoá is an urban lake. However, we have not discussed the influence of domestic animals in more detail as this metric was not measured. However, in the discussion (lines 381-384), we emphasized that the number of *A. sculptum* ticks may be related to the number of domestic animals, particularly dogs and cats (Mendes et al. 2019, Campos et al. 2020), rather than specifically to capybaras due to urbanization. It is worth noting that residents of luxurious houses often take their pets for leisurely walks, which may expose them to ticks.

It is also necessary to improve the quality of the figures.

Authors: Done.

References:
Campos SDE, Cunha NC, Machado CSC, Nadal NV, Seabra Junior ES, Telleria EL, et al. Spotted fever group rickettsial infection in dogs and their ticks from domestic–wildlife interface areas in southeastern Brazil. Braz J Vet Parasitol 2020; 29(1): e020219. <http://doi.org/10.1590/S1984-29612020012>.
Mendes JCR, Kmetiuk LB, Martins CM, Canavessi AMO, Jimenez T, Pellizzaro M, Martins TF, Morikawa VM, Santos APD, Labruna MB, Biondo AW. 2019. Serosurvey of Rickettsia spp. in cats from a Brazilian spotted fever-endemic area. Rev Bras Parasitol Vet 28:713–721.

Authors: Thanks, we included these references

**Reviewer #2: -** IT IS A DETAILED STUDY INCLUDING INFORMATION ABOUT ABIOTIC AND BIOTIC FACTORS.

Authors: Thanks.

BUT SOME ISSUES ARE NOT CLEAR, FOR EXAMPLE, HOW MANY DAYS A MONTH WAS THE SAMPLING EFFORT? WAS IT A MONTHLY SAMPLING?

Authors: Our sampling effort was 5 - 6 days: Set/21: 5 days, Nov/21: 5 days, Feb/22: 5 days, Apr/22: 6 days, Jun/22: 5 days, Sep/22: 5 days. (Line 105)

OTHER QUESTIONS ARE MARKED DIRECTLY IN THE TEXT.

Authors: Formatting changes in the manuscript file have been highlighted in yellow
